# A Robust Statistical Method for Association-Based eQTL Analysis

**DOI:** 10.1371/journal.pone.0023192

**Published:** 2011-08-09

**Authors:** Ning Jiang, Minghui Wang, Tianye Jia, Lin Wang, Lindsey Leach, Christine Hackett, David Marshall, Zewei Luo

**Affiliations:** 1 School of Biosciences, University of Birmingham, Birmingham, United Kingdom; 2 Laboratory of Population and Quantitative Genetics, Institute of Biostatistics, Fudan University, Shanghai, China; 3 BioSS, Invergowrie, Dundee, Scotland, United Kingdom; 4 Scottish Crop Research Institute, Invergowrie, Dundee, Scotland, United Kingdom; University of Texas School of Public Health, United States of America

## Abstract

**Background:**

It has been well established that theoretical kernel for recently surging genome-wide association study (GWAS) is statistical inference of linkage disequilibrium (LD) between a tested genetic marker and a putative locus affecting a disease trait. However, LD analysis is vulnerable to several confounding factors of which population stratification is the most prominent. Whilst many methods have been proposed to correct for the influence either through predicting the structure parameters or correcting inflation in the test statistic due to the stratification, these may not be feasible or may impose further statistical problems in practical implementation.

**Methodology:**

We propose here a novel statistical method to control spurious LD in GWAS from population structure by incorporating a control marker into testing for significance of genetic association of a polymorphic marker with phenotypic variation of a complex trait. The method avoids the need of structure prediction which may be infeasible or inadequate in practice and accounts properly for a varying effect of population stratification on different regions of the genome under study. Utility and statistical properties of the new method were tested through an intensive computer simulation study and an association-based genome-wide mapping of expression quantitative trait loci in genetically divergent human populations.

**Results/Conclusions:**

The analyses show that the new method confers an improved statistical power for detecting genuine genetic association in subpopulations and an effective control of spurious associations stemmed from population structure when compared with other two popularly implemented methods in the literature of GWAS.

## Introduction

Linkage disequilibrium (LD) based association mapping has received increasing attention in the recent literature [1]–[6] for its potential power and precision in detecting subtle phenotypic associated genetic variants when compared with traditional family-based linkage studies. Association mapping methods for the genetic dissection of complex traits utilize the decay of LD, the rate of which is determined by genetic distance between loci and the generation time since LD arose [7]. Over multiple generations of segregation, only loci physically close to the quantitative trait loci (QTL) are likely to be significantly associated with the trait of interest in a randomly mating population, providing great efficiency at distinguishing between small recombination fractions [8]. Despite this potential, many reported association studies have not been replicated or have resulted in false positives [9]–[10], commonly caused by ‘cryptic’ structure in population-based samples. Population structure, or population stratification [11], arises from systematic variation in allele frequencies across subpopulations, which can result in statistical association between a disease phenotype and marker(s) that have no physical linkage to causative loci [12]–[13], *i.e.* false positive or spurious associations. This gives rise to an urgent need for methods of adjusting for both population structure and cryptic relatedness occurring due to distant relatedness among samples with no known family relationships.

To avoid the problems raised from population stratification, family-based association studies have been proposed, such as the transmission-disequilibrium test (TDT), which compares the frequencies of marker alleles transmitted from heterozygous parents to affected offspring against those that are not transmitted [14]. In this design the ethnic background of cases and controls is necessarily matched, conferring robustness to the presence of population structure. However, TDT design requires samples from family trios, which are difficult to obtain compared to population based designs where a large sample is feasibly obtained. Moreover, increased genotyping efforts are required for TDT design to achieve the same power as population based design [15]–[16].

Numerous methods have been proposed to overcome the problems caused by population structure without the need for family based samples. Among the most widely used are the genomic control (GC) [17] and the structure association (SA) analysis [18]–[19]. In the former, inflation of the test statistic by population structure is estimated as a constant from unlinked markers in the genomic control group and then the test statistic will be adjusted from the estimate before being applied to infer the association. In the latter, unlinked markers are used to estimate the number of subpopulations from which the sample are collected, and then assign sample individuals to subpopulations. The former method considers an ideal but unrealistic situation of constant inflation factor for all markers, while in reality the influence of population structure on statistical inference of marker-trait association varies over genome locations [20]. For the SA method, it is computationally intensive to obtain accurate and reliable values for both the number of subpopulations in real datasets and to assign individual population membership. Alternative methods have been adopted to infer the subpopulation number, including Latent-Class model [21], mixture model [22] and a Bayesian model AdmixMap [23]. These methods share the assumption that associations among unlinked markers are the result of population structure and subpopulations are allocated to minimize these associations. This step depends critically upon the correct selection of a panel of markers to reflect population structure information. Price *et al.* [24] proposed a principal component analysis (PCA) based method, EIGENSTRAT, to model the ancestral difference in allele frequency and correct for population stratification by adjusting genotypes through linear regression on continuous axes of variation. While EIGENSTRAT provides specific correction for candidate markers, how to choose appropriate markers to infer population structure remains in question. In fact, prediction of the population structure may fail whenever the key assumption behind the structure prediction methods is violated.

Rather than using a panel of unlinked markers to exploit the cryptic population structure, a single null marker can be used to correct for bias of the test statistic in association studies. Wang *et al.* [25] suggested using a well-selected null marker to correct biases from population stratification on odds ratio estimation for a candidate gene within a logistic regression framework. They assumed a simplistic situation that the null marker had the same genotypic distribution as the candidate gene, which, however, was unknown in practice.

The expression quantitative trait locus (eQTL) analyses have recently shown that variation in human gene expression levels among individuals and also populations is influenced by polymorphic genetic variants [26]–[28]. The use of structured populations has meant that to detect the genetic variants accounting for differences in gene expression between subpopulations, GWAS had to be carried out separately for each subpopulation and the results subsequently compared. We present here a simple regression model of utilizing only one ‘control’ marker to remove the population structure effect in detecting LD between a marker and a putative quantitative trait locus. We first established the theoretical basis for selection and use of a control marker to correct for population structure and established a regression-based method for detecting the LD which is integrated with information of the control marker. We investigated the method for its efficiency to test the LD and to reduce false positives stemmed from population structure through intensive computer simulation studies and re-analysis of the gene expression (or eQTL) datasets collected from genetically divergent populations. The new method (**Method 1**) was compared with two alternative methods: single marker regression without population structure correction (**Method 2**) and multiple regression analysis with incorporation of known individual ancestry information (**Method 3**).

## Materials and Methods

### Method 1 (Regression analysis with correcting population structure)

The method analyzes a structured randomly mating population produced through instant admixture of two genetically divergent subpopulations. The proportion of subpopulation 1 in the mixed population is denoted by *m*. Let us consider three bi-allelic loci: one affects a quantitative trait (*Q*) while another two are polymorphic markers devoid of direct effect on the trait. We call, for convenience, one of the markers the test marker (*T*) which is to be tested for association with the QTL, and the other as control marker (*C*), assumed to be not associated with both the QTL and the test marker (*i.e.* the linkage disequilibrium *D* equal 0). Two alleles are denoted by *A* and *a* at the putative QTL, *T* and *t* at the test marker, and *C* and *c* at the control marker. Three genotypes at the QTL, *AA*, *Aa* and *aa*, are assumed to affect the quantitative trait by *d*, *h* and –*d* respectively. Trait phenotype of an individual (*Y*) is assumed to be normally distributed with mean depending on its genotype at the QTL and residual variance 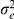. Genotypic values at the test marker and control marker are denoted by *X* and *Z*, which are the number of alleles *T* and *C* respectively. In subpopulation *i* (*i* = 1 or 2), the allelic frequencies of the QTL, test marker and control marker are denoted by 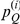, 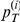 and 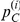 respectively, while the coefficients of linkage disequilibrium between any pair of the loci are denoted by 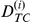, 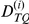 and 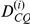. Table 1 illustrates probability distribution of joint genotypes at a test marker and a putative QTL in randomly mating populations together with genotypic values at the QTL and details for the parameterization can be found in Luo [29]. It is clear from Table 1 that the marker-QTL distribution can be fully characterized by the parameters defining population allele frequencies at the two loci and the coefficient of linkage disequilibrium between them. This provides the theoretical basis for statistical analyses developed below.

**Table 1:** Probability distribution of joint genotypes at a test marker and a putative QTL and genotypic values at the QTL.

Genotypes at QTL	*AA*			*Aa*			*aa*		
Marker genotypes	*TT*	*Tt*	*tt*	*TT*	*Tt*	*tt*	*TT*	*Tt*	*tt*
Probabilities	(*qQ*)^2^	2*q* ^2^ *Q*(1−*Q*)	*q* ^2^(1−*Q*)^2^	2 *q*(1−*q*)*QR*	2 *q*(1−*q*) (*Q*+*R*−2*QR*)	2 *q*(1−*q*) (1−*Q*)(1−*R*)	(1−*q*)^2^ *R* ^2^	2(1−*q*)^2^ *R*(1−*R*)	(1−*q*)^2^(1−*R*)^2^
Genotypic values at QTL	*μ*+*d*			*μ*+*h*			*μ*−*d*		

where *A* and *a* are segregating alleles at a putative QTL, *T* and *t* are alleles at the test marker locus. Allele frequency of *A* is *q*, allele frequency of *T* is *p*. *Q* and *R* are conditional probabilities of marker allele *T* given QTL allele *A* and *a* respectively, which are formulated as 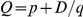 and 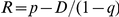 where *D* is the coefficient of linkage disequilibrium between the marker and QTL. *μ, d* and *h* are population mean, additive and dominance genic effects at the QTL.

#### Regression analysis correcting effect of population structure

For phenotype of a quantitative trait and each of the test markers, we fitted the following model: the genotype 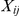 of individual 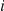 at the given marker locus 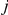 may be classified as one of three states: 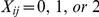 for homozygous rare, heterozygous and homozygous common alleles, respectively. For this model, we fitted a linear regression of the form for each genetic marker:
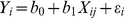
(1)where 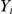 is phenotype for individual 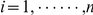, and 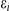 are independent normally distributed random variables with mean 0 and variance 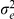. We have demonstrated that significance of the regression coefficient can be used to infer significance of LD between a polymorphic marker locus and a QTL in a single randomly mating population since the regression coefficient has a form of
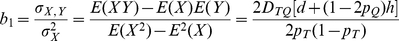
(2)
[29]. However, in a structured population, we note that the LD between a marker and a QTL is given by
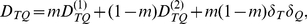
(3)
[30], where *m* is the proportion of subpopulation 1 in this mixed samples, the superscripts (1) and (2) refers to the subpopulations, 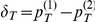 and 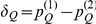. The covariance between the QTL and the test marker can be worked out as
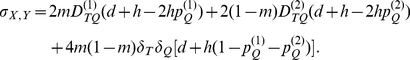
(4)Equations 3 and 4 show that the association between the QTL and test marker in a mixed population is the summation of (i) a linear combination of the associations between the two loci in each of the subpopulations (i.e. the genuine association due to LD between the two loci in each of the subpopulations), and (ii) a nonlinear component of the differences in allele frequencies between the two subpopulations (i.e. a spurious term of association). The objective of our analysis is to remove the spurious term by using a control marker ‘C’. If the control marker is neither in association with the QTL (i.e. 
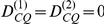
) nor with the test marker (
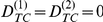
), then the covariance between control marker and QTL (or test marker) can be given by
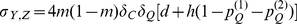
(5)

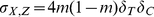
(6)In an admixed population, the control marker's allelic frequency is 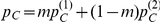. In a population with allelic frequency 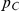 at the control marker locus, the expected and observed variances at the control marker are
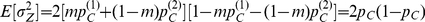
(7)

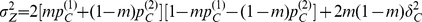
(8)where 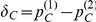. Thus, the difference between the expected and observed variances at the control marker indicates the existence of population structure,
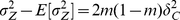
(9)The spurious term in the covariance in equation (4) can be completely corrected using a single control marker, as follows:
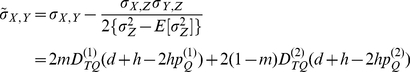
(10)Therefore, the regression coefficient calculated from
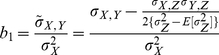
(11)would reflect correction for the population structure. The students *t*-test can be used to test for significance of the regression coefficient 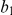. Standard error (se) of 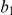 is given by
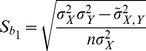
(12)Given the regression coefficients and their variances, the power of the regression analysis can be predicted from the probability [31]

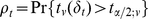
(13)where 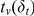 represents a random variable with non-central *t*-distribution with *v* degrees of freedom and non-centrality parameter 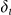 and 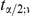 is the upper 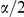 point of a central *t*-variable with the same degrees of freedom. The value of *v* equals *n*−3 and the non-centrality parameter is given by [31] as
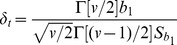
(14)where 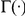 stands for a gamma function.

#### Selection of the control marker

In practice, we propose the following procedure to select the control marker for a given test marker. Firstly, any marker but the test marker would be candidate for the control marker if it has or is

- an autosomal location on different chromosomes from the test marker,
- less missing genotype data than a prior given proportion

For each marker passing the above screening, one calculates the expected and observed variances from
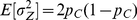
(15)

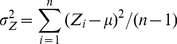
(16)where 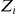 is the genotypic value of the candidate control marker (0, 1, 2) for individual 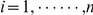, and 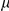 and 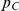 are the mean genotypic value across all individuals (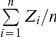 ) and the allelic frequency of this marker, respectively. It should be noted that equations (7) and (15) are the same and that equation (16) stands for the sampling variance of the control marker whose expectation is given by equation (8) in the presence of population structure. The control marker is the one with the maximum difference between observed and expected variances, which has the maximum ability to remove the spurious term in mixed populations and does not introduce bias in single population.

### Method 2 (Regression analysis without correcting population structure)

The method fits a simple regression model for detecting LD between the trait phenotype and a test marker as we proposed previously [29] and implemented in a recent population based eQTL analysis in [28], in which the regression coefficient has a form of
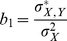
(17)with a standard error equal to
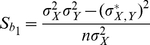
(18)where 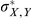 is the non-corrected covariance between test marker locus and the quantitative trait.

### Method 3 (multiple regression analysis)

The method regresses the trait phenotype on genotypic value of a test marker (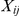 = 0, 1, 2) and the probability of membership to each constituent population *P_i_* (*i* = 1, 2 here) as described in the following multiple regression model
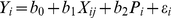
(19)where the 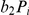 term reflects the population structure effect in mixed populations.

The regression coefficients are given by
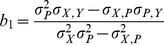
(20)

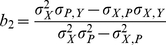
(21)and standard errors of the regression coefficients are formulated as
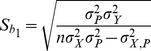
(22)

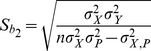
(23)according to [32]. Significance of association of the test marker with the quantitative trait can be tested through testing for significance of the regression coefficient *b_1_* by the Student *t*-test.

## Results

### Simulation study

To explore statistical properties and limitations of the methods described above, we developed and conducted a series of computation simulation studies. The simulation program mimics segregation pattern of genes at multiple marker loci and QTL in randomly mating natural populations in terms of simulation parameters defining allele frequencies, linkage disequilibria and population structure as illustrated in Table S1. The methods were detailed for simulating a population characterized the joint genotypic distribution at two loci and for sampling individuals from the simulated population [33]. Although the distribution involves only two loci, it is easy to extend to multiple loci because the two locus joint distribution can be easily converted into conditional (or transition) probability distribution of genotypes at one locus on that at another, and genotypes at multiple loci can be simulated as a Markov process governed by the conditional probability distribution. Of course, this will not undermine flexibility to specify any required linkage disequilibrium pattern among any loci. Subpopulations were independently generated and merged to produce the admixed population. In the present study, we were focused on 10 simulated populations defined by simulation parameters listed in Table S1.

Each simulation was repeated 100 times and simulation data was analyzed using the three different methods described above. We tabulated in Table 2 means and standard errors of 100 repeated regression coefficients and proportions of significant tests of the regression coefficients. It can be seen that **Methods 1** and **2** predicted the regression coefficients adequately in all simulated populations, but **Method 3** did so when all individuals were correctly allocated to their correct subpopulations. Listed in Table 2 were also proportions of significant tests of the regression in repeated simulations. It should be stressed that the proportion measures rate of false positive when the test marker and QTL were in linkage equilibrium such as in the first 4 simulated populations whilst it provides evaluation of an empirical statistical power for detecting the genetic association in populations 5 to 10. It is clear that the rate of false positive was properly controlled in association analysis with **Method 1**, and **Method 3** when all individuals were correctly allocated, and that LD between the test marker and QTL in populations 5–9 was tested significant by these methods with a high statistical power. In contrast, the simple regression analysis (**Method 2**) made a high proportion of false positive inference of the marker and QTL association when the LD was actually absent (populations 1–4) but failed to detect truly existing LD between the two loci (populations 5–9). The method is thus inappropriate to be used for genetic association analysis when population structure was present. Performance of **Method 3**, which incorporates membership of individuals to constituent populations as a covariate in multiple regression analysis, depends on the extent by which individuals are correctly allocated to their belonging populations. For example, the method lost its statistical power to detect the truly existing LD (populations 5–9) or made false positive inference of genetic association when on average a quarter of individuals under analysis were wrongly allocated to subpopulations (populations 1–4). These results show that the present method provides a powerful test for linkage disequilibrium between polymorphic markers and QTL and an effective control of population structure in the test.

**Table 2:** Means and standard errors of regression coefficients (*b*±se) and proportions (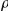 or 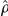) of statistical tests for significance of the regression coefficients from three methods.

Pop	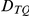	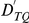	Method 1				Method 2				Method 3					
			Simulated		Predicted		Simulated		Predicted		Simulated				Predicted	
			*b* ± se	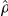	*b*	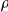	*b* ± se	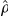	*b*	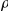	*b* ± se^a^	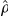 ^a^	*b* ± se^b^	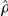 ^b^	*b* ^a^	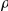 ^a^
1	0.04	0.00	−0.078±0.015	0.06	0.00	0.00	1.293±0.006	0.98	1.278	1.00	0.006±0.007	0.00	1.035±0.006	0.84	0.00	0.00
2	0.04	0.00	−0.087±0.015	0.07	0.00	0.00	1.162±0.006	0.97	1.163	0.98	−0.008±0.007	0.00	0.940±0.007	0.74	0.00	0.00
3	−0.09	0.00	0.015±0.008	0.00	0.00	0.00	−2.371±0.005	1.00	−2.368	1.00	0.006±0.007	0.00	−2.038±0.006	1.00	0.00	0.00
4	−0.09	0.00	0.005±0.011	0.00	0.00	0.00	−3.157±0.007	1.00	−3.157	1.00	−0.007±0.009	0.00	−2.725±0.008	1.00	0.00	0.00
5	0.02	0.05	0.965±0.021	0.48	0.828	0.55	−0.159±0.007	0.00	−0.166	0.00	0.997±0.006	0.85	0.082±0.007	0.00	0.994	0.91
6	0.04	0.07	1.086±0.008	0.86	1.062	0.92	0.130±0.007	0.00	0.125	0.00	1.280±0.006	1.00	0.375±0.007	0.01	1.274	1.00
7	0.05	0.08	1.341±0.008	0.98	1.325	1.00	0.333±0.007	0.01	0.331	0.01	1.593±0.006	1.00	0.597±0.007	0.14	1.59	1.00
8	0.05	0.08	1.260±0.006	0.99	1.249	0.99	0.313±0.007	0.01	0.312	0.01	1.503±0.006	1.00	0.572±0.007	0.13	1.499	1.00
9	0.04	0.08	1.307±0.014	0.92	1.234	0.99	−0.005±0.006	0.00	0.00	0.00	1.698±0.006	1.00	0.333±0.007	0.02	1.704	1.00
10	−0.04	0.00	0.008±0.009	0.01	0.00	0.00	−1.233±0.006	0.99	−1.234	0.99	−0.003±0.007	0.00	−0.995±0.007	0.80	0.00	0.00

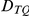
 and 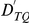 are the coefficients of LD between the marker and QTL in the simulated mixed population before and after correction for population structure respectively.

a predicted when all individuals were allocated to their correct subpopulations;

b predicted when half of all individuals were correctly allocated to their subpopulations but other half were randomly allocated to either of the two subpopulations. The predicted values were estimated from theoretical analysis, while the simulated values were estimated from the simulation studies.

Use of control markers in **Method 1** is the key underpinning for the method to be able to control influence of population structure in the genetic association test. To investigate effect of the control marker on efficiency of the association test, we explored performance of the method when population structure is actually absent or when different control markers are used in the presence of population structure. Table S2 shows predicted and observed proportions of significant tests of the disequilibrium between a test marker and a putative QTL in 10 simulation populations with (b) or without (a) population structure. The proportions were calculated from analyses with **Method 1** by using the control marker either with a constant allele frequency between two subpopulations or with varying allele frequencies. It demonstrates that the type I error is well controlled and the disequilibrium is efficiently detected by the method using a control marker even when population structure does not actually exist (a). In addition, when population structure is present (b), the method bears a high chance to make a false positive inference and to lose its detecting power if the control marker selected to be implemented in the analysis has a small difference in allele frequency between the subpopulations. However, the risk can be effectively controlled and the reduced power can be recovered when using the control marker with a large allele frequency difference. All these suggest that implementation of control markers with a non-trial difference in allele frequency will not cause any significant problem of false positive/negative inference when population stratification is actually not existent. In presence of population structure, we propose selection of a marker with largely divergent allele frequencies as the control marker.

### Gene expression and genotype datasets

The gene expression and SNP datasets were collected from Epstein-Barr virus (EBV) transformed lymphoblastoid cell lines of unrelated individuals of European-derived (CEU, 60 Europeans), and Asia-derived (CHB+JPT, 41 Chinese and 41 Japanese). The datasets were originally developed by Spielman et al [28] to explore population specified gene expression and genetic control of the population specified gene expression, and were downloaded from http://www.ncbi.nlm.nih.gov/geo (Gene Expression Omnibus: GSM5859). The expression arrays were analyzed using the Affymetrix MAS 5.0 software and the hybridization intensity was log_2_-transformed into expression phenotype. The study focused on 4,197 genes that are expressed in lymphoblastoid cell lines. Of the 4,197 genes, 1,097 were detected to be significantly differentially expressed between the CEU and CHB+JPT samples (*t*-test, 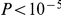; 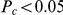, Sidak correction) [34]. SNP data scored on the 60 CEU, 41 CHB and 41 JPT samples were obtained from the International HapMap Project (release 19,). All markers with an allele frequency of ≥5% were included, giving more than 2.2 million and 2.0 million common SNP markers for the CEU samples and CHB+JPT samples respectively. Comparison between the CEU and CHB+JPT samples provided genotype data for 1,606,182 unique SNP markers among all 142 individuals (60 CUE and 82 CHB+JPT samples).

We selected and re-analysed the gene expression and SNP datasets in the present study for several reasons. Firstly, these samples were collected from the populations whose genetical diversification was well verified [35]–[37], and make a typical example which the method is designed for. Secondly, gene expression phenotype bears a wide spectrum of genetic controls from *cis* to *trans* regulation and different levels of heritability. Some of these quantitative phenotypes show population specified expression or heterogeneity of underlying genetics. These enable the method to be tested under different genetic backgrounds. Finally, re-analysis of the same datasets recently published allows a direct comparison of analysis with the method developed in the present study with that implemented in the published analysis.

### Validation of population structure

**Figure 1:**
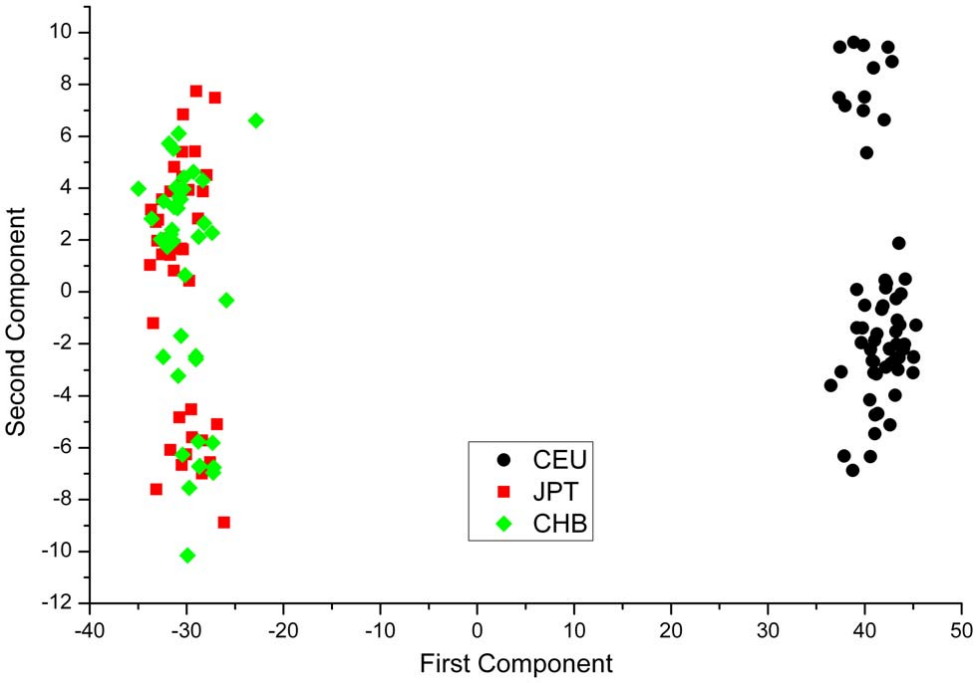
The first 2 Principal Components from PCA of 142 mixed HapMap Project human samples. The first and second principal components explained 60.77% and 1.34% of total variability respectively.

In 2005, The International HapMap Project reported that the CHB and JPT samples' allele frequencies were generally very similar, but different to the allele frequencies of CEU samples (Figure S1). We first explored deviation in genotypic distribution at each of nearly 2 million SNP markers from the Hardy-Weinberg equilibrium (HWE) within CEU and CHB+JPT samples separately and in mixed of the two samples by using both Pearson's chi-squared test and Fisher's exact test. To account for the multiple tests, we set the significant different level at 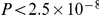 (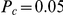 after Sidak correction). The analyses did not detect any of the SNP markers whose genotypic distribution showed significant deviation from HWE in either of the two samples. However, when all CEU and CHB+JPT samples were merged together there were approximately 3,000 markers scattered across all autosomes deviating significantly from the HWE expectation (2911 markers from Pearson's chi-squared test, consistent with 3011 markers from Fisher's exact test). These analyses show that the CEU and CHB+JPT samples can be recognized to be collected from genetically divergent random matting populations and that a mixed of them represents an example of samples from these populations. Population structure in the mixed sample was visualized as a score plot of the first two principal components built on the 2911 SNP markers, which explained a total of 62% of variability of the marker data (Figure 1).

### Genome-wide association eQTL analysis

We implemented the three methods described above to perform association mapping of eQTL using the gene expression and SNP marker datasets. The analysis was carried out on the CEU and CHB+JPT samples separately or jointly. An eQTL in the present analysis was defined as an independent peak in the p-value profile across a given chromosome. Peaks occurring within 5 Mb of adjacent peaks were taken as a single eQTL peak because of insufficient evidence to declare the existence of multiple eQTL peaks over such narrow intervals [38]. The eQTL location was defined as the location within the peak with the smallest p-value. To account for the large number of tests, we set the significance level at nominal 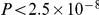 (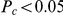 after Sidak correction), a conservative level also used previously [28], [34]. A *cis*-regulated eQTL was operationally defined by the presence of significant association with a SNP in the region 500 kb upstream of the start of the transcript to 500 kb downstream of the 3′ end; otherwise, the eQTL was classified as *trans-*acting. Table 3 summarizes the number of eQTL detected by the three methods (**Method 1** developed in the present study, **Method 2** the simple regression analysis employed by Spielman et al in [28], and **Method 3** the multiple regression analysis) from the Europe derived, Asia derived samples and their mixed respectively. It can be seen that the eQTL analysis results from the CEU and CHB+JPT samples are comparable between **Method 1** and **2** in terms of the number of detected eQTLs and estimated locations of these eQTLs, suggesting a comparable predictability of the two methods in the absence of population structure. In the mixed sample, 64% of eQTL detected by the multiple regression analysis (**Method 3**) with use of full population membership information can be recovered by the method developed in the present study (**Method 1**), confirming the predictability of the latter in the presence of the population structure. We explored the predictability of **Method 3** when individuals were randomly assigned to the Europe derived sample (CEU) with probability of 58% or to the Asia derived sample (CHB+JPT) otherwise. The analysis showed that only 12% (240/1,975) of eQTL detected by the method with the partial population membership information was consistent with those detected by the same method with the full membership information, suggesting that the predictability of the method depends heavily on certainty of the membership information and that the method may generate a large proportion of false positives when the information is not complete.

**Table 3:** The number of eQTLs detected by three different methods (**Methods 1, 2, 3** or **M1, 2, 3 accordingly**) or detected common between two of these methods from the CEU, CHB+JPT and their mixed samples.

The number of eQTLs per expression trait	The CEU samples			The CHB+JPT samples			The mixed CEU and CHB+JPT samples				
	M1	M2	M1+2	M1	M2	M1+2	M1	M3	M1+3	M3^a^	M3+3^a^
1	280	312	**225**	263	255	**209**	206	251	**145**	398	**89**
2	58	57	**33**	43	41	**25**	16	13	**5**	136	**1**
3	20	21	**10**	13	16	**7**	2	7	**2**	97	**0**
4	10	16	**6**	8	6	**4**	2	2	**1**	72	**0**
5	4	4	**1**	5	6	**2**	0	0	**0**	48	**0**
6	3	1	**1**	1	3	**1**	0	0	**0**	37	**0**
7	3	3	**1**	0	2	**0**	0	0	**0**	22	**0**
8	0	2	**0**	1	0	**0**	1	0	**0**	22	**0**
9	2	1	**1**	0	0	**0**	0	1	**0**	14	**0**
> = 10	19	22	**5**	6	7	**1**	2	2	**1**	1,111	**1**
Total eQTLs	1,009	1,149	**912**	633	670	**554**	296	354	**226**	1,975	**240**
*cis*-eQTLs	21	22	**21**	48	49	**48**	51	58	**51**	618	**53**
*trans*-eQTLs	988	1127	**891**	585	621	**506**	245	296	**175**	1,339	**187**

M3^a^ is for Method 3 when individuals were randomly assigned to the Europe derived sample (CEU) with probability of 58% or to the Asia derived sample (CHB+JPT) otherwise.

**Figure 2:**
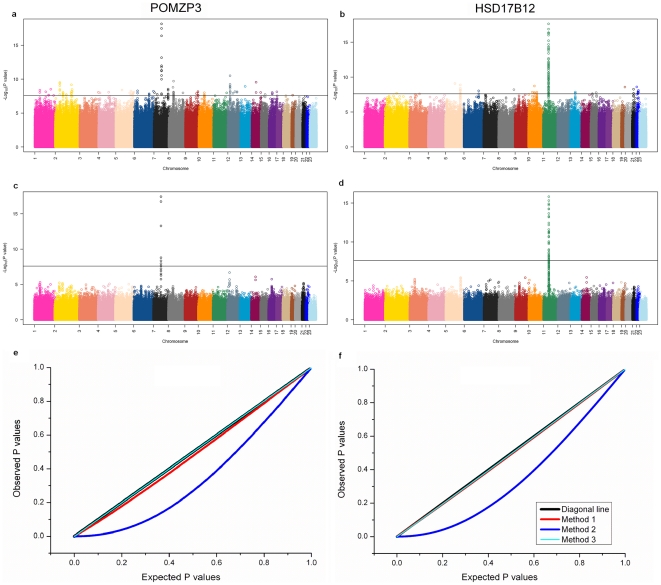
Manhattan plots for the genome-wide eQTL analysis of two genes POMZP3 and HSD17B12; Quantile-quantile (QQ) plots to compare the distributions between expected and observed p-values. Plots show score (−log_10_ p-value) for all SNPs by physical position for POMZP3 and HSD17B12 respectively based on simple linear regression (**Method 2**, a and b) and corrected linear regression (**Method 1**, c and d) in 142 mixed population samples.

The POMZP3 and HSD17B12 (on the human chromosome 7 q11.23 and chromosome 11 q11.2 respectively) are two well-characterized and *cis*-regulated genes [26], [28], [38]–[41]. Although all the three methods considered here were able to detect the previously identified *cis*-regulators from the three samples, there were a large number of spurious association signals predicted from the simple regression analysis (**Method 2**) with the mixed sample (Figure 2: a and b, respectively). It is clear that these spurious associations were effectively removed in the analysis with **Method 1**, reflecting the effectiveness of the latter in controlling the false positives (Figure 2: c and d, respectively). In the mixed samples, **Method 1** was able to reveal 296 significant eQTL, 51 of which were *cis*-regulators (Table 3). Firstly, the cis- eQTL predicted here include all the 11 *cis*-acting regulators reported by Spielman et al. [28] who performed the simple regression analysis (**Method 2**) in the CEU and CHB+JPT samples separately. In addition to 16 previously detected *cis-* acting factors, **Method 1** detected 35 novel cis- eQTL and all the eQTL explained 20∼70% of variability in expression of the genes regulated (Table S3). We compared the 245 *trans*-regulators detected by our method from the mixed sample against the Gene Ontology (GO) Molecular Function annotation database (http://www.geneontology.org/) and found that 101 (42%) *trans*-eQTLs predicted were mapped into the category of transcriptional factors, 82 (33%) *trans*-regulators played a role in signal pathway activity. In total, 75% *trans-*regulators predicted by the present method were previously known to play a role in gene regulation. All these reveal a significantly improved statistical power of the present method in detecting the true genetic associations.

**Figure 3:**
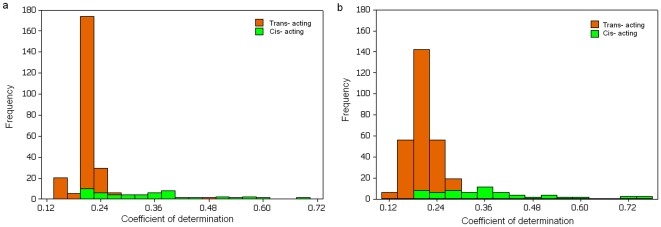
Histograms of coefficient of determination for eQTLs from 142 mixed sample set. a for **Method 1** and b for **Method 3**.

It is interesting to note that the number of *cis*- eQTL detected from the mixed samples is larger than that from the component samples separately whilst a much larger number of *trans*- eQTL are detected in the component samples than in their mixed. This observation may reflect the fact that an increase in size of the mixed sample has enhanced the statistical power to detect cis- eQTL and thus led to an increased number of cis- eQTL detected. However, if linkage disequilibria between genes regulated and their trans- regulators are in opposite directions between different populations, the LD may be counter-balanced in the merged population, and thus decrease the number of the trans- eQTL to be detected. Despite a relatively small number of *cis* eQTLs detected, the *cis-*regulated effects were generally stronger than those in *trans*, with about 14% (7/51) *cis*-acting eQTL having a determination coefficient 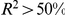 (Figure 3), consistent with findings in human and mice [38], [42]–[44].

## Discussion

Linkage disequilibrium based association mapping has been advocated as the method of choice for identifying chromosomal regions containing disease-susceptibility loci or loci affecting other complex quantitative traits of interest [45]. However, it is well known that the presence of population structure can result in false positive inference of genetic association between a test marker and trait loci. Various methods have been proposed in the literature to tackle this problem [19], [21]–[23], [46] and many of them have heavily depended on adequate prediction of the population structure [18], [24]. Efficiency of the methods is thus largely affected by adequacy of population structure prediction. It has been shown that adequate prediction of population structure is in fact not a feasible task [47]. On the other hand, it is obvious that effect of the population stratification on association tests may vary across different regions of the genome [6]–[8]. Thus, the methods designed to correct for the stratification caused spurious associations through adjusting the test statistic by subtracting a constant inflation in the statistic may not perfectly reflect this observation [10], [25]. To address these problems, we have proposed here a statistical method for correcting for stratification confounding effect in LD-based QTL mapping. The method extends the idea of using control markers to correct for background effect on a statistical test for significance of QTL at any given genome position in linkage-based QTL mapping analysis [48] and enables the effect of population stratification in the LD-based QTL analysis to be adjusted at a local basis. We presented here a simple but effective method to determine the control marker and demonstrated that incorporation of control markers would not cause any significant statistical problem even though population structure does actually not exist.

The new method developed in this study is tested and compared with other most popularly implemented methods in the literature of genetic association studies through intensive computer simulation studies and analysis of large scale and high quality gene expression and SNP datasets for mapping expression QTL. These analyses strongly support outperformance of the new method for its significantly improved statistical power to detect genuine LD between any polymorphic markers and putative trait loci and its effectiveness in controlling spurious association due to population stratification. Worthwhile, although the multiple regression analysis based on a mixed linear model does also provide a control of the influence of population stratifications, its efficiency depends heavily on accuracy of prediction of the population structure and on accurate allocation of individuals' membership to the constituent populations. Any bias in the structure prediction and uncertainty in the membership allocation may lead to severe consequence on its analytical efficiency. It has been argued that several factors may substantially influence or even disable the prediction of population structure [49]–[50]. Therefore, the method virtually avoids the need for sophisticated prediction of population ancestry of individuals and, in turn, effectively controls any bias embedded with the prediction. The method was designed for modeling and analyzing samples collected from different ethnical (or ecological) cohorts (or populations) with or without a clear clue about their genetic diversity. This is a very popular practice in many GWAS analyses, particularly with human samples [28], [51]–[54].

Wang et al has proposed use of a single null marker to correct for population structure in a candidate gene based association analysis using case and control samples [25]. In their settings, the null marker was fitted as a dichotomous variable in parallel to the test candidate gene in a logistic regression model, and the influence of population structure on the association test at the candidate gene was adjusted by subtracting the regression coefficient associated with the null marker from the coefficient associated with the gene. Question rises to the parallel formulation: which is the major effect to be tested in the model? In contrast, our method was developed upon a rigorous population genetics model in which contributions of three different loci (i.e. the test marker, QTL and control marker) to the linkage disequilibrium pattern are properly formulated. The method is thus more appropriate for population based association studies. Although theoretical analysis was built on a single marker test, the idea and principle of the method could be extendable to the haplotype-based association mapping which uses information from multiple marker loci [55]–[56]. This is because the population confounding term is linearly attached to the main disequilibrium terms in the covariance between the test polymorphism and trait effect (Equation 3). Our goal is to remove the confounding term from the covariance and, thus form of the main disequilibrium terms either in genotype at an individual marker locus or in haplotypes at multiple marker loci will not affect the way to correct for the confounding term. Although the method was presented for two genetically divergent populations, the overall pattern of LD between any test marker and trait locus in their admixed population may become theoretically more complicated when the admixture involves more than two populations. Before having invested more theoretical investigation to the problem, we would suggest to merge those genetically less divergent objects together as we did in the present analysis with the Chinese and Japanese samples and to correct for the stratification raised from between the most divergent populations such as the European derived and the Asia derived samples.

## Supporting Information

Figure S1 **Comparison of allele frequencies between populations for all SNP markers genotyped in the International HapMap Project.** The colour in each bin represents the number of SNPs that display each given set of allele frequencies.
(TIF)

Table S1 **Parameters defining two subpopulations that are merged to produce admixed populations.**
(DOC)

Table S2 **Predicted and observed proportions of significant tests of linkage disequilibrium between a test marker and a putative QTL in different simulation populations from Method 1 in which the control marker implemented into the analyses had either (a) no population structure, and has a constant allele frequency difference of 0.4 at control marker locus or (b) population structure exist, and has varied allele frequency differences at control marker locus.**
(DOC)

Table S3 **The 51 cis-eQTLs predicted by Method 1 from the mixed sample.**
(DOC)
