# Supplementary figures and images for: A Robust Statistical Method for Association-Based eQTL Analysis
Source: PLoS One. 2011 Aug 9;6(8):e23192. doi: 10.1371/journal.pone.0023192 (PMC3153488; doi:10.1371/journal.pone.0023192)

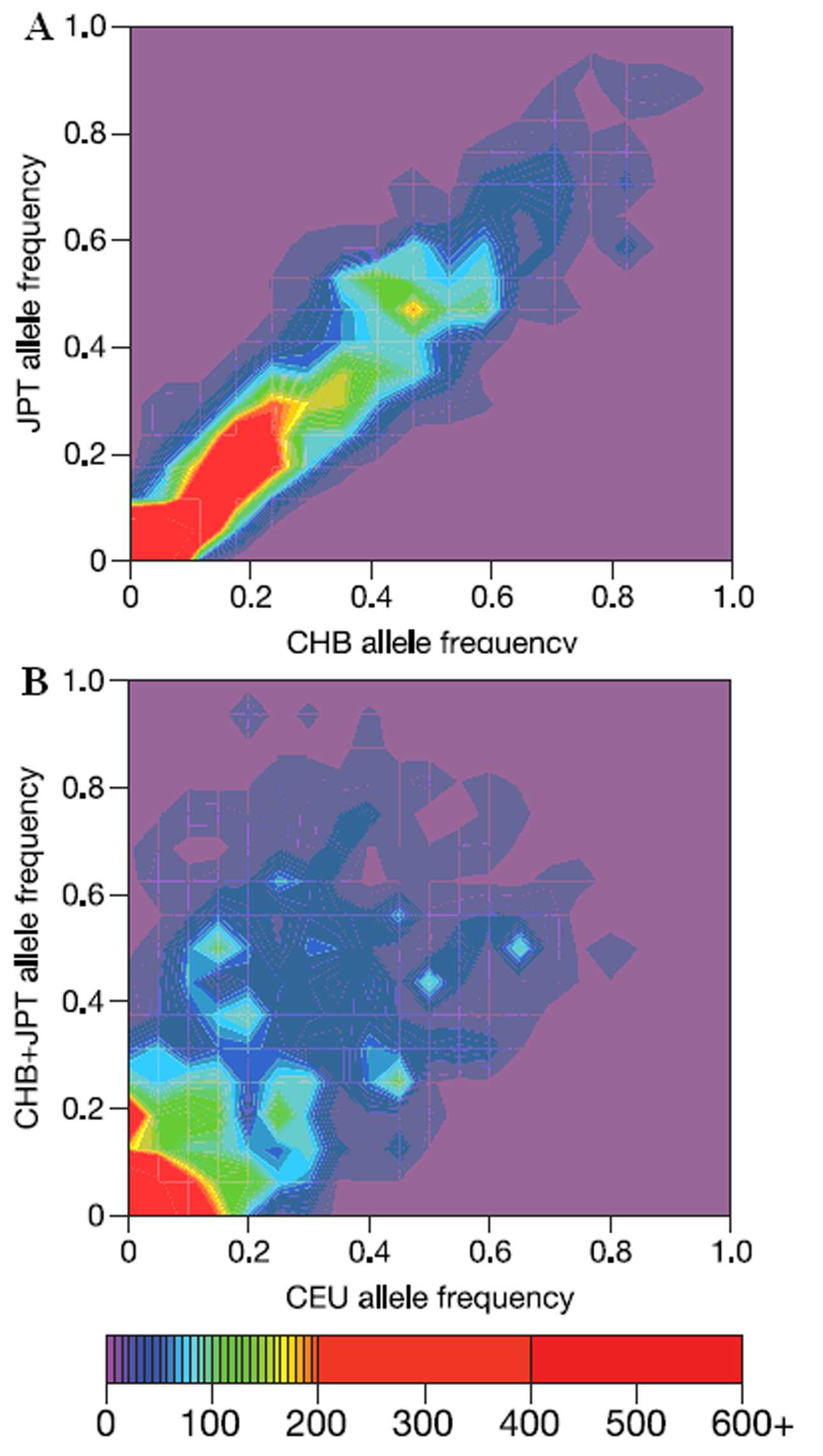

Supplement: Figure S1 — Comparison of allele frequencies between populations for all SNP markers genotyped in the International HapMap Project. The colour in each bin represents the number of SNPs that display each given set of allele frequencies. (TIF) [file pone.0023192.s001.tif]
