# Supplementary material for: A Robust Statistical Method for Association-Based eQTL Analysis
Source: PLoS One. 2011 Aug 9;6(8):e23192. doi: 10.1371/journal.pone.0023192 (PMC3153488; doi:10.1371/journal.pone.0023192)
Supplement: Table S1 — Parameters defining two subpopulations that are merged to produce admixed populations. (DOC) [file pone.0023192.s002.doc]

Table S1 Parameters defining two subpopulations that are merged to produce admixed populations.

| Pop. | *n* | *m* | *h^2^* | *Φ* | $p_{Q}^{(1)}$ | $p_{T}^{(1)}$ | $p_{C}^{(1)}$ | $D_{TQ}^{(1)}$ | $p_{Q}^{(2)}$ | $p_{T}^{(2)}$ | $p_{C}^{(2)}$ | $D_{TQ}^{(2)}$ |
| --- | --- | --- | --- | --- | --- | --- | --- | --- | --- | --- | --- | --- |
| 1 | 500 | 0.5 | 0.1 | 0 | 0.3 | 0.4 | 0.7 | 0.00 | 0.7 | 0.8 | 0.3 | 0.00 |
| 2 | 500 | 0.5 | 0.1 | 0.5 | 0.7 | 0.7 | 0.3 | 0.00 | 0.3 | 0.3 | 0.7 | 0.00 |
| 3 | 500 | 0.5 | 0.1 | 0 | 0.2 | 0.8 | 0.2 | 0.00 | 0.8 | 0.2 | 0.8 | 0.00 |
| 4 | 500 | 0.5 | 0.2 | 0.5 | 0.2 | 0.8 | 0.2 | 0.00 | 0.8 | 0.2 | 0.8 | 0.00 |
| 5 | 500 | 0.5 | 0.1 | 0 | 0.3 | 0.7 | 0.5 | 0.05 | 0.7 | 0.4 | 0.8 | 0.05 |
| 6 | 500 | 0.5 | 0.1 | 0.5 | 0.3 | 0.7 | 0.3 | 0.08 | 0.7 | 0.4 | 0.8 | 0.05 |
| 7 | 500 | 0.5 | 0.1 | 0 | 0.3 | 0.7 | 0.3 | 0.08 | 0.7 | 0.4 | 0.8 | 0.08 |
| 8 | 500 | 0.5 | 0.1 | 0.5 | 0.3 | 0.7 | 0.2 | 0.08 | 0.7 | 0.4 | 0.8 | 0.08 |
| 9 | 500 | 0.5 | 0.1 | 0 | 0.3 | 0.7 | 0.7 | 0.08 | 0.7 | 0.3 | 0.3 | 0.08 |
| 10 | 500 | 0.5 | 0.1 | 0 | 0.3 | 0.7 | 0.3 | 0.08 | 0.7 | 0.3 | 0.8 | -0.08 |

*n* is the sample size, *m* is the proportion of subpopulation 1 in the admixture, *h^2^* is QTL heritability defined in an equilibrium population with QTL allelic frequency *p*=0.5, *Φ* is the dominance ratio at the QTL, $p_{Q}^{(i)}$, $p_{T}^{(i)}$and$p_{C}^{(i)}$are respectively the allelic frequencies at QTL, test marker and control marker in the *i*-th subpopulation (*i*=1,2), and $D_{TQ}^{(i)}$is the coefficient of linkage disequilibrium between QTL and test marker in *i*-th subpopulation.
