# Supplementary material for: A Robust Statistical Method for Association-Based eQTL Analysis
Source: PLoS One. 2011 Aug 9;6(8):e23192. doi: 10.1371/journal.pone.0023192 (PMC3153488; doi:10.1371/journal.pone.0023192)
Supplement: Table S2 — Predicted and observed proportions of significant tests of linkage disequilibrium between a test marker and a putative QTL in different simulation populations from Method 1 in which the control marker implemented into the analyses had either (a) no population structure, and has a constant allele frequency difference of 0.4 at control marker locus or (b) population structure exist, and has varied allele frequency differences at control marker locus. (DOC) [file pone.0023192.s003.doc]

Table S2 Predicted and observed proportions of significant tests of linkage disequilibrium between a test marker and a putative QTL in different simulation populations (without population stratification (a) and with population stratification (b) ) from Method 1 in which the control marker implemented into the analyses had either (a) a constant allele frequency difference of 0.4 or (b) varying allele frequency differences.

| a | | | | | | | | b | | | | | | | |
| --- | --- | --- | --- | --- | --- | --- | --- | --- | --- | --- | --- | --- | --- | --- | --- |
| Pop | $p_{Q}^{(1)}=p_{Q}^{(2)}$ | $p_{T}^{(1)}=p_{T}^{(2)}$ | $D_{TQ}^{'}$ | Estimated | | Predicted | | $p_{Q}^{(1)}=p_{T}^{(2)}$ | $p_{T}^{(1)}=p_{Q}^{(2)}$ | $\left\vert p_{C}^{(1)}-p_{C}^{(2)}\right\vert$ | $D_{TQ}^{'}$ | Estimated | | Predicted | |
|  |  |  |  | $\hat{b}\pm se$ | $\hat{\rho}$ | *b* | $\rho$ |  |  |  |  | $\hat{b}\pm se$ | $\hat{\rho}$ | *b* | $\rho$ |
| 1 | 0.50 | 0.50 | 0.00 | 0.003±0.007 | 0.00 | 0.00 | 0.00 | 0.30 | 0.70 | 0.10 | 0.00 | -0.408±0.042 | 0.45 | 0.000 | 0.00 |
| 2 | 0.55 | 0.50 | 0.00 | 0.001±0.007 | 0.00 | 0.00 | 0.00 | 0.30 | 0.70 | 0.20 | 0.00 | 0.066±0.035 | 0.25 | 0.000 | 0.00 |
| 3 | 0.55 | 0.45 | 0.00 | -0.001±0.007 | 0.00 | 0.00 | 0.00 | 0.30 | 0.70 | 0.40 | 0.00 | 0.049±0.014 | 0.04 | 0.000 | 0.00 |
| 4 | 0.60 | 0.45 | 0.00 | 0.019±0.008 | 0.00 | 0.00 | 0.00 | 0.30 | 0.70 | 0.60 | 0.00 | -0.005±0.010 | 0.00 | 0.000 | 0.00 |
| 5 | 0.60 | 0.40 | 0.00 | 0.024±0.009 | 0.01 | 0.00 | 0.00 | 0.30 | 0.70 | 0.80 | 0.00 | 0.006±0.006 | 0.00 | 0.000 | 0.00 |
| 6 | 0.50 | 0.50 | 0.05 | 0.894±0.007 | 0.66 | 0.894 | 0.74 | 0.30 | 0.70 | 0.10 | 0.08 | 0.781±0.028 | 0.34 | 1.325 | 1.00 |
| 7 | 0.55 | 0.50 | 0.05 | 0.899±0.007 | 0.67 | 0.894 | 0.74 | 0.30 | 0.70 | 0.20 | 0.08 | 1.292±0.029 | 0.70 | 1.325 | 1.00 |
| 8 | 0.55 | 0.45 | 0.05 | 0.878±0.007 | 0.65 | 0.886 | 0.72 | 0.30 | 0.70 | 0.40 | 0.08 | 1.385±0.013 | 0.94 | 1.325 | 1.00 |
| 9 | 0.60 | 0.45 | 0.06 | 1.058±0.007 | 0.87 | 1.052 | 0.93 | 0.30 | 0.70 | 0.60 | 0.08 | 1.325±0.006 | 1.00 | 1.325 | 1.00 |
| 10 | 0.60 | 0.40 | 0.06 | 1.475±0.011 | 0.85 | 1.46 | 0.91 | 0.30 | 0.70 | 0.80 | 0.08 | 1.321±0.005 | 1.00 | 1.325 | 1.00 |
