# Supplementary material for: A Robust Statistical Method for Association-Based eQTL Analysis
Source: PLoS One. 2011 Aug 9;6(8):e23192. doi: 10.1371/journal.pone.0023192 (PMC3153488; doi:10.1371/journal.pone.0023192)
Supplement: Table S3 — The 51 cis-eQTLs predicted by Method 1 from the mixed sample. (DOC) [file pone.0023192.s004.doc]

Table S3 The 51 cis-eQTLs predicted by **Method 1** from the mixed sample.

| NUM | Gene | cis-SNP ID | SNP position | P-value | $R^{2}$ | Reference |
| --- | --- | --- | --- | --- | --- | --- |
| **1** | **UGT2B17** | **rs3100645** | **Chr4:69806739** | **2.22E-16** | **0.38** | **[28]** |
| **2** | **POMZP3** | **rs2005354** | **Chr7:75856016** | **3.87E-28** | **0.58** | **[28] , [38]** |
| **3** | **PEX6** | **rs2395943** | **Chr6:42987528** | **3.13E-17** | **0.40** | **[28]** |
| **4** | **PSPHL** | **rs10243293** | **Chr7:55583849** | **2.25E-10** | **0.25** | **[28] , [38]** |
| **5** | **CSTB** | **rs2838386** | **Chr21:44080386** | **4.95E-12** | **0.29** | **[28] , [38]** |
| **6** | **DNAJD1** | **rs2281778** | **Chr13:41395977** | **2.64E-13** | **0.32** | **[28]** |
| **7** | **AP3S2** | **rs4932265** | **Chr15:88153061** | **1.24E-11** | **0.28** | **[28]** |
| **8** | **HSD17B12** | **rs1061810** | **Chr11:43842243** | **4.77E-16** | **0.37** | **[28], [38]** |
| **9** | **NUBP2** | **rs1065663** | **Chr16:1779024** | **2.02E-09** | **0.22** | **[28]** |
| **10** | **B4GALT1** | **rs10124479** | **Chr9:33126233** | **5.94E-10** | **0.24** | **[28]** |
| **11** | **TPP2** | **rs1887355** | **Chr13:100933170** | **1.09E-08** | **0.21** | **[28]** |
| **12** | **IRF5** | **rs12155080** | **Chr7:128212697** | **7.33E-18** | **0.41** | **[38]** |
| **13** | **CHI3L2** | **rs942694** | **Chr1:111082865** | **1.20E-09** | **0.23** | **[38]** |
| **14** | **CPNE1** | **rs12480408** | **Chr20:34950229** | **1.56E-19** | **0.44** | **[38]** |
| **15** | **CTSH** | **rs10400902** | **Chr15:76947435** | **6.90E-15** | **0.35** | **[38]** |
| **16** | **GSTM2** | **rs366631** | **Chr1:109551199** | **8.83E-26** | **0.54** | **[38]** |
| 17 | DFNA5 | rs12700538 | Chr7:24379328 | 1.35E-09 | 0.23 | - |
| 18 | HEBP2 | rs2076273 | Chr6:138684210 | 5.80E-09 | 0.21 | - |
| 19 | EVI2A | rs2107359 | Chr17:29842786 | 1.29E-08 | 0.20 | - |
| 20 | CRYZ | rs10890142 | Chr1:74549064 | 5.40E-09 | 0.21 | - |
| 21 | PARP4 | rs7317850 | Chr13:22792037 | 1.59E-09 | 0.23 | - |
| 22 | RRM1 | rs10767857 | Chr11:4132198 | 4.92E-13 | 0.31 | - |
| 23 | RPL31 | rs12472882 | Chr2:101267759 | 3.70E-23 | 0.50 | - |
| 24 | HLA-DPB1 | rs9277463 | Chr6:33100194 | 5.46E-30 | 0.60 | - |
| 25 | TSG101 | rs1395320 | Chr11:18512504 | 4.37E-12 | 0.29 | - |
| 26 | DDX42 | rs1043127 | Chr17:62264581 | 4.50E-17 | 0.39 | - |
| 27 | MEST | rs12672246 | Chr7:129671295 | 6.04E-09 | 0.21 | - |
| 28 | AMFR | rs2440468 | Chr16:56196080 | 9.57E-15 | 0.35 | - |
| 29 | GSTM3 | rs1332018 | Chr1:109581699 | 1.24E-08 | 0.21 | - |
| 30 | ECD | rs6480700 | Chr10:74446293 | 7.26E-10 | 0.24 | - |
| 31 | MTRR | rs326123 | Chr5:7929599 | 2.17E-13 | 0.32 | - |
| 32 | RABGGTA | rs3940231 | Chr14:22738491 | 7.23E-16 | 0.37 | - |
| 33 | BACH1 | rs733610 | Chr21:29576646 | 2.66E-15 | 0.36 | - |
| 34 | GSTM1 | rs366631 | Chr1:109551199 | 2.72E-27 | 0.57 | - |
| 35 | SLC7A7 | rs12884337 | Chr14:21263242 | 1.15E-11 | 0.28 | - |
| 36 | TAP2 | rs241448 | Chr6:32843645 | 2.11E-16 | 0.38 | - |
| 37 | APOBEC3B | rs17000581 | Chr22:37608815 | 2.47E-09 | 0.22 | - |
| 38 | NT5C2 | rs10883824 | Chr10:104477484 | 4.01E-16 | 0.38 | - |
| 39 | HBS1L | rs12663447 | Chr6:135297596 | 2.08E-24 | 0.52 | - |
| 40 | HLA-DQB1 | rs9275141 | Chr6:32697538 | 5.55E-15 | 0.35 | - |
| 41 | BTN3A2 | rs9366653 | Chr6:26462226 | 1.06E-16 | 0.39 | - |
| 42 | TLR1 | rs3924112 | Chr4:38692990 | 1.03E-13 | 0.32 | - |
| 43 | MXRA7 | rs1014390 | Chr17:75310009 | 1.29E-13 | 0.32 | - |
| 44 | CD47 | rs6768207 | Chr3:109345802 | 2.88E-11 | 0.27 | - |
| 45 | GLT8D1 | rs736408 | Chr3:52792702 | 5.38E-09 | 0.21 | - |
| 46 | TIMM13 | rs3848633 | Chr19:2360880 | 1.47E-17 | 0.40 | - |
| 47 | POLR1D | rs9512760 | Chr13:25971662 | 1.23E-08 | 0.21 | - |
| 48 | SMUG1 | rs3136375 | Chr12:52867203 | 8.83E-13 | 0.30 | - |
| 49 | POLR1E | rs10758432 | Chr9:37478009 | 7.49E-11 | 0.26 | - |
| 50 | ERAP2 | rs2548540 | Chr5:96304251 | 4.95E-39 | 0.70 | - |
| 51 | IPP | rs12091503 | Chr1:45521162 | 6.67E-17 | 0.39 | - |
